# Knee Joint Motion Detection Based on Demodulation of Overlapping Spectrum Using Fiber Bragg Grating Sensor

**DOI:** 10.3390/s26113341

**Published:** 2026-05-25

**Authors:** Linlin Fan, Lingzhen Yang, Juanfen Wang, Weijie Ding, Huizhi Ren, Chao Zhou

**Affiliations:** 1 College of Physics and Optoelectronics, Taiyuan University of Technology, Taiyuan 030024, China; 2 Shanxi Key Laboratory of Precision Measurement Physics, Taiyuan University of Technology, Taiyuan 030024, China; 3 Key Laboratory of Advanced Transducers and Intelligent Control System, Ministry of Education, Taiyuan University of Technology, Taiyuan 030024, China; 4 Department of Physics, Xinzhou Normal University, Xinzhou 034000, China

**Keywords:** overlap spectrum, fiber laser, fiber Bragg grating, demodulation, knee joint motion

## Abstract

This study proposes a knee joint motion detection method based on overlapping spectrum demodulation using fiber Bragg grating (FBG) technology. A flexible FBG encapsulated with polydimethylsiloxane (PDMS) is attached to the joint surface. Axial strain in the FBG sensor is generated due to the bending and extension movements of the joint, which leads to a central reflection wavelength shift of the FBG sensor. The overlapping spectrum between the FBG reflection and the output of a tunable fiber laser is related to the wavelength shift of the FBG. The variation is expressed as the changes in reflected optical power received by an optical power meter. It transforms complex spectral analysis into intuitive optical power measurement for demodulating the reflected wavelength of the FBG sensor. The relationship between the optical power of the overlapping spectrum and wavelength shift of the FBG induced by joint motion is theoretically and experimentally analyzed. The real-time demodulation of joint motion is realized based on this relationship. Experimental results demonstrate that the system exhibits good repeatability in monitoring knee joint motion. The performance and practical potential of the system are evaluated through a quantitative comparison with existing techniques and an analysis of its current limitations.

## 1. Introduction

Joint diseases have become a major issue seriously threatening the health of the elderly and are a significant cause of disability as the world population ages [1,2]. Studies have shown various effective approaches to solve joint health issues, such as early identification [3,4], early diagnosis [5] and effective intervention [6,7], which can significantly reduce mortality rates and disability rates [8,9,10]. Joint motion detection and rehabilitation assessment are key technologies in motion analysis and rehabilitation medicine. Assessment of rehabilitation is currently very subjective and relies heavily on the experience and skills of the physiotherapists and the doctors. Although traditional detection methods such as optical markers [11], computed tomography (CT) [12,13], magnetic resonance imaging (MRI) [14] and inertial measurement units (IMUs) [15,16] are relatively mature, they still have several limitations in practical applications including susceptibility to environmental interference, bulky equipment, high costs, marker point recognition, poor repeatability, significant hysteresis, nonlinear response [17], electromagnetic interference and difficulties in long-term wearing [18,19]. Most existing joint monitoring systems are large and structurally complex. Some systems also require a skilled operator and laboratory setup to acquire the data or demodulate joint-related information.

**Figure 1:**
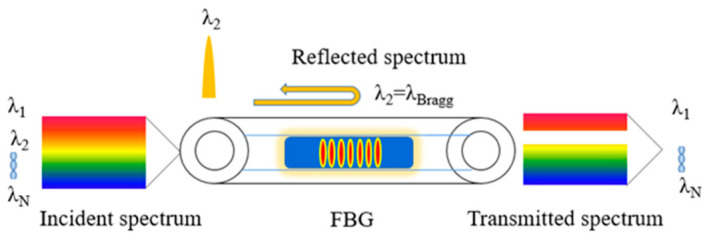
The working principle of the FBG.

With the continuous development of optical technology, FBGs have shown great potential in biomedical sensing due to advantages such as immunity to electromagnetic interference, high sensitivity, compact size, easy multiplexing, and good biocompatibility [20,21]. FBGs are versatile sensors used in a wide range of applications, and their compatibility with embedded sensing structures makes them especially suitable for joint sensing. The working principle of an FBG-based sensor system relies on the reflected Bragg signal as shown in Figure 1. FBG sensors are well suited to dynamic strain sensing due to the linear relationship between Bragg wavelength shift and applied strain. In recent years, flexible FBG-based sensors have demonstrated remarkable progress in monitoring respiration [22,23], heart rate [24], joint motion [25,26], and surface deformation [27], providing a promising technological solution for long-term, wearable human motion monitoring.

According to the sensing theory of the FBG [28], the shift in the central wavelength of the FBG caused by strain follows the photo-elastic effect. When broadband light (incident spectrum) propagates through the FBG, only a specific wavelength satisfying the Bragg condition is strongly reflected (reflected spectrum), while the remaining wavelengths are transmitted. The Bragg wavelength in FBG is given by

(1) $$\lambda_{B}=2n\Lambda$$

where Λ is the grating period and n is the effective refractive index of the fiber core. The shift in the Bragg wavelength in the FBG and the change in strain exhibit a linear relationship within a certain range. Δλ is the change in wavelength caused by the change in external parameters. When only the strain effect of the FBG is considered, the formula is as follows:

(2) $$\Delta \lambda_{B}=\lambda_{B}\left(1-P\right)*\varepsilon$$

(3) $$\Delta \lambda_{B}/\lambda_{B}=\left(1-P\right)*\varepsilon =K*\varepsilon$$

The Bragg wavelength of the FBG is $\lambda_{B}$, P is the photo-elastic coefficient of the optical fiber, and ε is the strain applied on the FBG. The equation indicates that there is a linear relationship between the variation in the Bragg wavelength of the FBG and the strain induced by external factors, as shown in Equation (2). The ratio K=1−P in Equation (3) expresses the strain effect on an optical fiber. It corresponds to a change in the grating spacing and the strain-optic-induced change in the refractive index. In this context, the K acts as the strain sensitivity factor of the FBG, and the linear coefficient represents the sensing sensitivity of the FBG.

Signal demodulation is a key challenge in FBG sensing research. In addition, the cost of FBG sensing systems is dependent on the demodulation technology. Traditional demodulation schemes based on Amplified Spontaneous Emission (ASE) sources typically rely on high-resolution optical spectrum analyzers (OSAs). While these instruments offer ease of setup and exceptional resolution [29], they are prohibitively expensive and have limited suitability for continuous monitoring. Generally, the demodulation techniques of signals are classified into two main categories: interference techniques and filtering techniques. Interferometric demodulation is typically implemented using interferometer configurations, primarily the Mach–Zehnder interferometer [30,31] and an unbalanced Michelson interferometer [32]. Filtering techniques typically include tunable Fabry–Pérot filters [33] and matched FBG filters [34]. Although these methods offer reduced cost and complexity compared to OSA-based solutions, they are still constrained by a narrow dynamic range, poor tunability, and inadequate sensitivity. Furthermore, their reliance on specialized optical components limits their portability and suitability for dynamic monitoring [35,36]. Other demodulation techniques include cavity ring-down and cross-correlation, which are used for strain sensing and precise localization [37]. Meanwhile, tunable fiber lasers can enhance the measurement range and accuracy of FBG sensing systems compared to other existing approaches.

Therefore, there are some key challenges involving signal demodulation as well as reducing the cost in the application of FBG sensors. The existing wavelength demodulation methods (such as spectrometer, matching grating method, etc.) usually rely on high-precision optical devices, resulting in complex systems and high costs, which limits their wide application. Furthermore, pulsed light sources are employed in most FBG-based sensing systems. In practical applications, the pulse width of the light source is subject to certain requirements and constraints in the time domain, which will impact the multiplexing capability and the cost of the system. However, the advantages offered by tunable fiber lasers can overcome these limitations, reduce system costs, and enhance the demodulation performance of FBG sensing, as demonstrated in the research.

In the paper, an overlapping spectrum demodulation scheme based on an FBG and a tunable wavelength fiber laser is proposed for joint motion detection. The method exploits the correlation between changes in the overlapping spectral region and the reflected optical power, which transforms conventionally complex spectral analysis into intuitive power measurement. The reflected optical power can be directly measured by an optical power meter. The linear relationship between the power and reflected wavelength is obtained by the curve fitting technique to demodulate knee joint motion signals. Compared to conventional FBG interrogation schemes, the proposed method eliminates the need for expensive optical spectrum analyzers and offers a simplified demodulation architecture, thereby enhancing cost-effectiveness and demonstrating strong potential for biomedical sensing applications.

## 2. Experiment Setup

**Figure 2:**
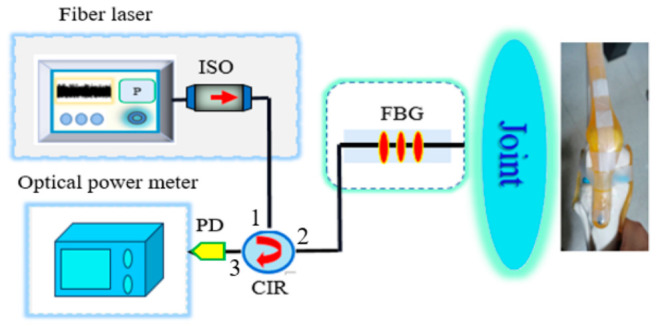
The experimental setup for joint motion detection.

The proposed FBG sensing setup for joint motion detection is shown in Figure 2. The configuration consists of a fiber laser, FBG sensor, optical power meter and a knee joint. In previous work of our research group, fiber lasers have been experimentally demonstrated [37,38]. A tunable fiber grating as a filter is installed in the fiber laser. The filter is used to keep the output spectral width of the fiber laser the same as the central reflection spectral width of the FBG sensor. The fiber laser enters into the sensing system by an optical circulator (CIR), and the FBG sensing unit is attached to the knee joint. Standard optical fibers were used to connect the FBG sensor to the stationary measurement system. The optical isolator (ISO) ensures the unidirectional transmission of the fiber laser. The light power reflected by the FBG sensor can be measured using an optical power meter. The optical power variation of the FBG sensor was monitored using an Optical Power Meter (PM20, Thorlabs), which utilizes an InGaAs detector (800–1700 nm) with a measurement range of −50 dBm to +23 dBm (10 nW to 20 mW) and an uncertainty of ±0.25 dB. The actual detected signals consistently fell within the 0.05–0.3 mW range, validating that the instrument’s specifications fully cover the operating power level of our experimental setup. The system demodulates the wavelength shift of the FBG by monitoring the variation in the total optical power of the reflected light.

The joint motion monitoring system operates as follows: A fiber laser serves as the optical source, emitting continuous-wave light into the sensing system. The light first passes through the ISO to prevent back-reflections and ensure laser stability. It is then directed into port 1 of the CIR and exits from port 2. The light travels through an optical fiber and transmits to the FBG sensor attached to the joint, inducing its characteristic reflection. When the joint moves, the induced strain causes a shift in the Bragg wavelength of the FBG. The narrowband light reflected from the FBG travels back along the fiber and re-enters the circulator at port 2. According to its operating principle, the circulator routes this reflected light from port 2 to port 3. This signal is subsequently processed and measured by an optical power meter. Consequently, real-time monitoring of the detected optical power variations enables effective demodulation of the dynamic strain on the FBG sensor and, therefore, of the joint motion.

### 2.1. Fabrication of the FBG Sensor

**Figure 3:**
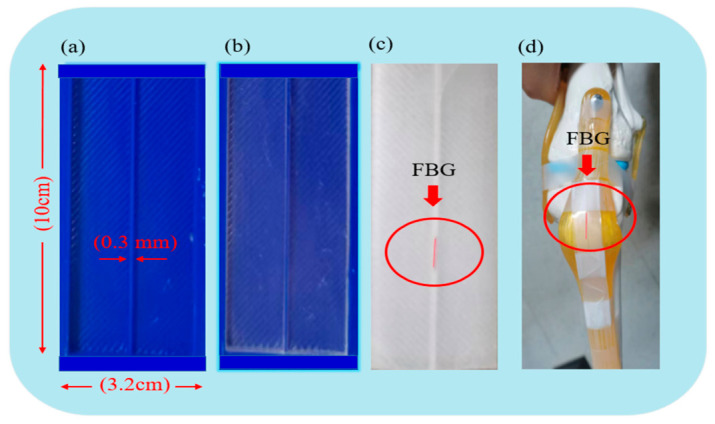
The FBG sensor. (**a**) Mold, (**b**) PDMS, (**c**) embedded FBG sensor, (**d**) knee joint.

The FBG is encapsulated with PDMS and functions as the sensing unit of system. PDMS is composed of a polydimethylsiloxane base liquid and a curing agent mixed at a ratio of 10:1. A mold with a raised bottom was designed by three-dimensional (3D) printing as shown in Figure 3a. The length of the mold is 10 cm and the width is 3.2 cm. The middle raised part is designed to be 0.3 mm in diameter combining with the single-mode fiber cladding diameter. This mold is used to fabricate the PDMS substrate for encapsulating FBG sensors. PDMS was placed in a pre-prepared mold for curing at room temperature (maintained at 25 °C) for 24 h to achieve the desired shape as shown in Figure 3b. The FBG encapsulated with PDMS possesses a flexible structure and can be placed on (or removed from) the knee very easily as shown in Figure 3c. It is attached to the knee joint mold in Figure 3d. The purpose of the fabrication is to provide mechanical protection for the FBG, ensure biocompatibility and supply a flexible, conformable substrate that facilitates robust attachment to the joint.

### 2.2. FBG Strain Pre-Stretching

In the experiment, the FBG is placed on the micro-displacement platform, and it is pre-stretched to make its initial center wavelength greater than its own center wavelength. The pre-stretching of the FBG was analyzed experimentally and strain response of the FBG was calibrated. As wavelength-modulated devices, FBG sensors enable environmental variations to be captured by monitoring shifts in the Bragg wavelength.

**Figure 4:**
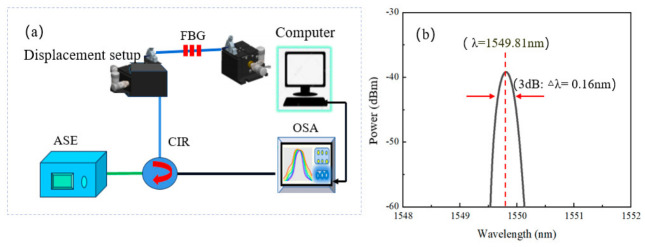
(**a**) The setup for pre-stretching FBG, (**b**) the reflection spectrum of the FBG.

The strain response of the FBG was calibrated through a pre-stretching process. The experimental setup is shown in Figure 4a, where a manually tunable micro-displacement platform was used to apply strain to the FBG. This platform has a spatial resolution of 0.001 mm and a strain control accuracy of 1 με, ensuring precise control of strain increments. A broadband ASE light source with a wavelength range of 1546–1564 nm was used to illuminate the FBG, and the FBG reflection spectrum was measured by an optical spectrum analyzer (OSA) with a resolution of 0.02 nm. The reflection spectrum of the FBG is shown in Figure 4b, featuring a central wavelength of 1549.81 nm, a 3 dB bandwidth of 0.16 nm, and a reflectivity of 10%. The strain applied to the FBG was controlled by the micro-displacement platform. The evolution of the central wavelength was monitored during a complete cycle in which the strain was incrementally increased from 0 με to 5000 με and then decreased back to 0 με in steps of 500 με. A stabilization time of 5 min was allowed after each strain step to ensure a steady state of the FBG.

**Figure 5:**
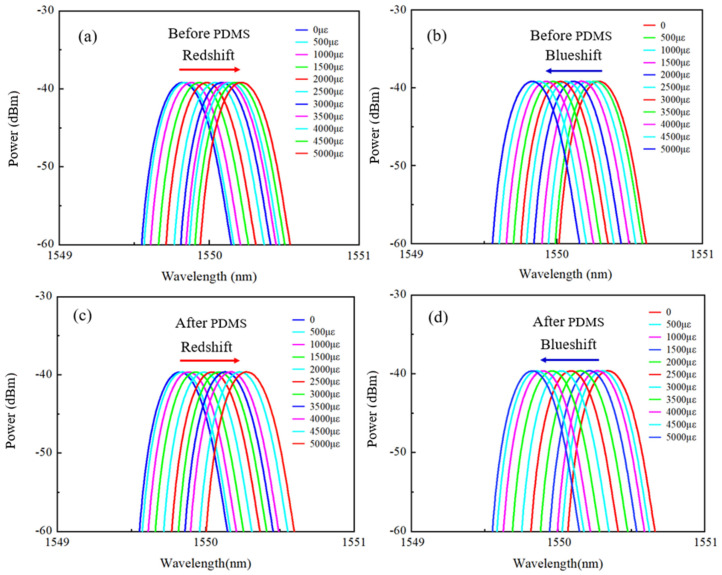
FBG wavelength shift with strain. (**a**) The redshift before PDMS, (**b**) the blueshift before PDMS, (**c**) the redshift after PDMS, (**d**) the blueshift after PDMS.

This pre-stretching analysis was performed separately on the FBG before encapsulation and after PDMS encapsulation, with the results presented in Figure 5. As shown in Figure 5a,c, the reflected wavelength of the FBG exhibits a redshift with increasing strain while the spectral shape remains consistent. Correspondingly, when the strain is reduced back to zero, a blueshift in the central reflected wavelength of the FBG is observed as shown in Figure 5b,d. The highly consistent spectral shapes of the FBG under various strain levels throughout the pre-stretching process demonstrate its excellent repeatability and long-term stability.

**Figure 6:**
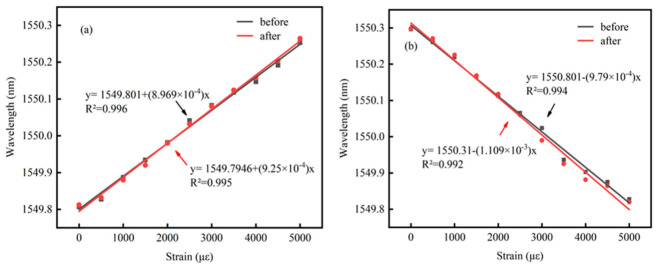
Wavelength shift with strain. (**a**) The redshift of the central wavelength of the FBG, (**b**) the blueshift of the central wavelength of the FBG.

Figure 6 presents the relationship between the central reflection wavelength shift of the FBG and the applied strain. During pre-stretching, the reflected wavelength of the FBG exhibits a linear redshift of approximately 0.514 nm within the strain range of 0 to 5000 με as shown in Figure 6a. The strain sensitivity of the FBG (before PDMS), derived from the slope of the linear fit, was measured to be 8.97 × 10^−4^ nm/µε and 9.26 × 10^−4^ nm/µε after PDMS encapsulation. A corresponding wavelength blueshift of approximately 0.507 nm is observed when the strain is reduced from 5000 με back to zero as depicted in Figure 6b. The sensitivity of the FBG before PDMS is 9.79 × 10^−4^ nm/με and 1.109 × 10^−3^ nm/με after PDMS. The packaged FBG sensor exhibits a strain sensitivity of 1.109 × 10^−3^ nm/με (≈1.109 pm/με), close to the theoretical value of 1.2 pm/με. The mean squared error (MSE) of the linear fit is 0.001, indicating good linearity and measurement repeatability. This minimal deviation may be attributed to minor strain transfer losses of the encapsulation layer or subtle variations in the bonding interface. According to the linear relationship (with a coefficient of determination R^2^ > 0.99) between the central wavelength of the FBG and the strain, a wide range of strains can be detected with a smaller wavelength shift using FBG.

Pre-stretching is applied to the FBG sensor to relieve residual strain within the grating and homogenize internal stress, thereby establishing a stable baseline for strain measurement. This process gradually transitions the fiber Bragg grating from a partially stretched state into a uniformly strained and well-coupled working condition, thereby enhancing the strain transfer efficiency. The strain response is calibrated via the pre-stretching process. PDMS encapsulation preserves the strain-sensing validity of the FBG sensor while providing biocompatibility, flexibility, and mechanical protection.

### 2.3. Demodulation Principle

**Figure 7:**
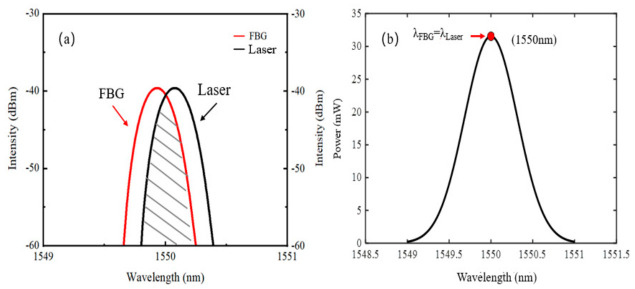
(**a**) Spectrum of laser and reflected spectrum of the FBG, (**b**) the simulation of the voltage with the shift in wavelength of the FBG.

The demodulation principle of the sensing system is based on the variation of overlap area between the spectrum of the FBG and fiber laser. Figure 7a indicates the fiber laser spectrum $P\left(\lambda \right)$ and the reflection spectrum $R\left(\lambda \right)$ of the FBG employed to interrogate the FBG. The mathematical expressions of these spectra ($P\left(\lambda \right)$ and $R\left(\lambda \right)$) are adopted with reference to the definitions in [37]:

(4) $$P\left(\lambda \right)=P_{0}\exp\left[-\alpha_{1}{\left(\lambda -\lambda_{0}\right)}^{2}\right],\;\alpha_{1}=\frac{4\ln2}{a^{2}}$$

(5) $$R\left(\lambda \right)=R_{B}\exp\left[-\alpha_{2}{\left(\lambda -\lambda_{B}\right)}^{2}\right],\;\alpha_{2}=\frac{4\ln2}{b^{2}}$$

where $\lambda_{0}$ and $\lambda_{B}$ correspond to the central wavelengths of the incident laser spectra and FBG separately, $P_{0}$ is incident optical power on the detector from the FBG reflection, $R_{B}$ corresponds to the FBG reflectivity at its central wavelengths, and $\alpha_{1}$ and $\alpha_{2}$ are their full width at half maximum (FWHM). The FBG is equivalent to a narrowband filter, and the fiber light with incident wavelengths equal to the central reflection wavelength of the FBG is reflected back when the laser is injected into the sensing system. The optical power reflected by an FBG is expressed as the overlap integral between the incident optical spectrum and the spectral reflectivity of the FBG across all wavelengths. Therefore, the total reflected optical power of the FBG can be obtained by evaluating the following integral:

(6) $$P=\int_{-\infty }^{+\infty }R\left(\lambda \right)P\left(\lambda \right)d\lambda$$

Substituting mathematical expressions of $P\left(\lambda \right)$ and $R\left(\lambda \right)$ into Equation (6) and integrating the results over wavelength, we can obtain

(7) $$P=P_{0}R_{B}\int_{-\infty }^{+\infty }\exp\left[-\alpha_{1}{\left(\lambda -\lambda_{0}\right)}^{2}-\alpha_{2}{\left(\lambda -\lambda_{B}\right)}^{2}\right]d\lambda$$

To solve this integral analytically, we first expand the quadratic terms in the exponent and rearrange the coefficients; the exponent can be written as quadratic function of λ:

(8) $$-\left(\alpha_{1}+\alpha_{2}\right)\lambda^{2}+2\left(\alpha_{1}\lambda_{0}+\alpha_{2}\lambda_{B}\right)\lambda -\left(\alpha_{1}\lambda_{0}^{2}+\alpha_{2}\lambda_{B}^{2}\right)$$

Completing the square for this quadratic expression, let $A=\alpha_{1}+\alpha_{2}$. The exponent is then reformulated into the standard Gaussian form:

(9) $$-A{\left[\lambda -\frac{\alpha_{1}\lambda_{0}+\alpha_{2}\lambda_{B}}{A}\right]}^{2}+\frac{{\left(\alpha_{1}\lambda_{0}+\alpha_{2}\lambda_{B}\right)}^{2}}{A}-\left(\alpha_{1}\lambda_{0}^{2}+\alpha_{2}\lambda_{B}^{2}\right)$$

Further simplifying the constant term outside the square bracket, we have

(10) $$\frac{{\left(\alpha_{1}\lambda_{0}+\alpha_{2}\lambda_{B}\right)}^{2}-\left(\alpha_{1}+\alpha_{2}\right)\left(\alpha_{1}\lambda_{0}^{2}+\alpha_{2}\lambda_{B}^{2}\right)}{\alpha_{1}+\alpha_{2}}=\frac{\alpha_{1}\alpha_{2}}{\alpha_{1}+\alpha_{2}}{\left(\lambda_{0}-\lambda_{B}\right)}^{2}$$

Thus, the integral is transformed into a standard Gaussian function. Utilizing the Gaussian integral identity, $\int_{-\infty }^{\infty }e^{-Au^{2}}du=\sqrt{\pi /A}$. Consequently, based on the integral properties of the product of Gaussian functions, the analytical solution for the optical power is finally derived as

(11) $$P=P_{0}R_{B}\sqrt{\frac{\pi }{\alpha_{1}+\alpha_{2}}}\exp\left[-\frac{\alpha_{1}\alpha_{2}}{\alpha_{1}+\alpha_{2}}{\left(\lambda_{0}-\lambda_{B}\right)}^{2}\right]$$

The core theoretical model for wavelength demodulation of the FBG sensing system is based on Equation (11) in this paper. The relationship between optical power (P) and central wavelength ($\lambda_{B}$) of the FBG incurred by knee joint motion is established. This equation indicates that the total reflected optical power of the FBG is given by the overlap spectrum integral between the incident laser and the FBG reflection. Specifically, the total optical power is proportional to the incident optical power and reflectivity of the FBG, and it is related to the center wavelength mismatch $\left(\lambda_{0}-\lambda_{B}\right)$ of the two spectra. When strain is applied on the FBG, it causes a variation in the wavelength shift of the FBG. Under the condition of an invariant wavelength of the incident fiber laser, a variation in wavelength ($\lambda_{B}$) can change the overlap area between the incident laser spectrum and the reflection spectrum of the FBG. The power of the light reflected by the FBG sensor is related to the overlapping shadow area as shown in Figure 7a. The optical power incident on the detector is determined by the overlapping area of the two waveforms. Therefore, the wavelength shift caused by strain is converted into detectable changes in optical power. The overlapping region between the reflection spectrum of the FBG and the incident light spectrum of the fiber laser remains unchanged when they are not affected by environmental changes. The overlapping area of the spectrum will change when either of them is disturbed by an external factor. Therefore, the overlapping spectrum can be used to detect any physical quantity that can cause a shift in the FBG reflection spectrum. This demodulation scheme transforms complex spectral analysis into an intuitive optical power measurement, enabling the detection of dynamic strain. The wavelength shift of each FBG is converted into an optical power variation. Consequently, the wavelength shift caused by strain can be demodulated by real-time monitoring of the light power reflected by the FBG according to the theoretical relationship described in Equation (11). When all other system parameters are known, the optical power signal is translated into precise wavelength data, which subsequently enables the quantification of mechanical deformation.

In order to verify the feasibility of the demodulation approach, the simulation of the sensing system is carried out. Figure 7b illustrates the simulated relationship between the detected optical power and the reflected wavelength shift of the FBG based on the theoretical model according to Equation (8). In the simulation, the fiber laser wavelength ($\lambda_{L\mathrm{aser}}$) was set to 1550 nm, the peak reflectivity of the FBG is 0.1, and the spectral width parameters both of the fiber laser and the FBG reflection are set to 0.1. The values listed are exemplary and used for generating the theoretical curve. In practice, they should be determined or calibrated according to the specific experimental setup. The horizontal and vertical axes represent the FBG wavelength (varying with applied strain) and the corresponding optical power, respectively. The obtained response curve presents a characteristic Gaussian-like profile. The peak occurs when the laser spectrum matches the reflection spectrum of FBG ($\lambda_{L\mathrm{aser}}=\lambda_{FBG}$), indicating maximum spectral overlap, and then the optical power decreases symmetrically as the two spectra detune with each other. The relationship between the two is generally nonlinear, but it can be approximated as linear within a small range near the peak, which is suitable for high-sensitivity demodulation.

The feasibility of the intensity-based demodulation scheme employed in this work is demonstrated theoretically based on the characteristic curves obtained through theoretical derivation. Thus, by monitoring the real-time variation in optical power with a power meter, it can be directly and correlated to the wavelength shift of the FBG. This correlation allows for the calculation of dynamic strain and the monitoring of joint motion. Therefore, this theoretical result provides a foundational basis for our subsequent experimental procedures.

## 3. Experimental Results and Discussion

**Figure 8:**
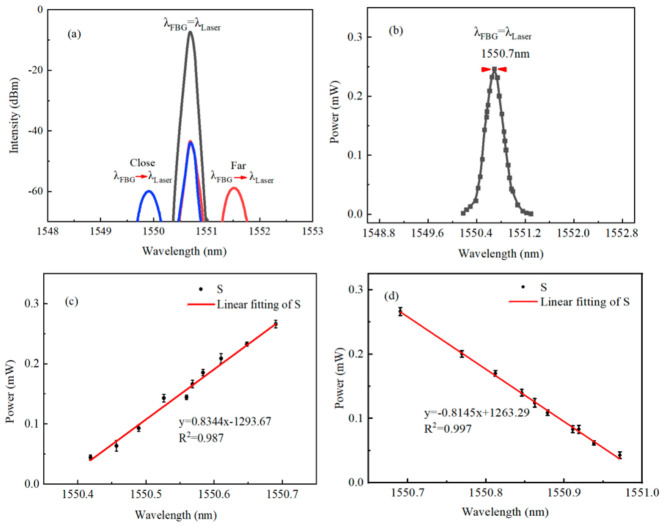
(**a**) Spectral demodulation of joint motion, (**b**) the change in the power with the wavelength of the FBG experimentally, (**c**) the change in the power with 0.8344 μW/nm, (**d**) the change in the power with −0.8145 μW/nm.

In the experiment, the motion of the knee joint model was tested to verify the optical power demodulation in the FBG sensing system. Induced strain in the FBG due to bending or extension movement of the knee joint was achieved. Before joint motion, the laser wavelength was tuned to 1550.70 nm, which is 0.89 nm away from the reflection wavelength of the FBG. The strain induced by the joint motion caused the reflection wavelength of the FBG to shift. The shift in the FBG reflection wavelength changes the overlapping spectral area between the FBG and laser as shown in Figure 8a. The reflection spectrum shifts dynamically relative to the fixed laser spectrum. The two spectra first gradually approach each other, and then they partially overlap. Subsequently, they completely coincide and finally they separate from each other again. The detected optical power is directly influenced by variations in the overlapping spectral region, whose value is directly measured by an optical power meter. The FBG wavelength was determined from the optical power values recorded during each knee bending and straightening cycle. Figure 8b shows the experimental verification of the relationship between reflected power and FBG reflection wavelength. It indicates variations in the overlap area of the FBG and laser. The peak power represents the state where the spectral overlap area is maximum and the reflected light from the FBG is strongest, indicating that the laser wavelength aligns with the center wavelength of the FBG. When the wavelength deviates from the center, the power decreases symmetrically, indicating a reduction in the overlapping region and a corresponding drop in optical power, which is consistent with the fundamental principle of overlapping spectrum demodulation [39]. The experiment involves periodic flexion and extension movements of the knee joint. By demodulating the wavelength shifts of the reflected light from the FBG in different power levels, continuous tracking of the joint movement can be achieved throughout the entire testing period.

Figure 8c shows the response of the FBG sensor in the range of 1550.4–1550.7 nm. The FBG sensor presents a good linear response to the change in wavelength and with a sensitivity of 0.8344 μW/nm (with uncertainty: ±0.0034 μW/nm). Figure 8d shows the response of the FBG sensor in the range of 1550.7–1551.0 nm and with a sensitivity of −0.8145 μW/nm (with uncertainty: ±0.0013 μW/nm). This indicates the FBG sensing system has great stability of operation scope in the range of 1550.4–1551.0 nm. The optical power at each central wavelength of the FBG mentioned above was the average of ten measurements. The error bars of the ten measurements are shown in Figure 8c,d, and the standard deviation of the optical power measured is kept within 0.006 mW. The small error bars in the graph suggest low variability across repeated measurements at each data point and high measurement precision. This indicates the sensing system has great stability. The sensing system can have different wavelength demodulation regions when the fiber laser wavelength is set to other values. The proposed method can reduce the need for high-performance hardware or equipment.

The knee kinematics is represented by two stages: flexion and extension. In the experiment, the optical power values corresponding to the FBG wavelength shifts induced by the knee joint movements were recorded. The reflected wavelength of the FBG corresponding to each optical power value was demodulated based on the theoretical model of Equation (8), and the motion within three cycles was analyzed. The optical power corresponding to each wavelength was measured by an optical power meter, and the shifted wavelength of the FBG was subsequently calculated based on the established linear relationship.

**Figure 9:**
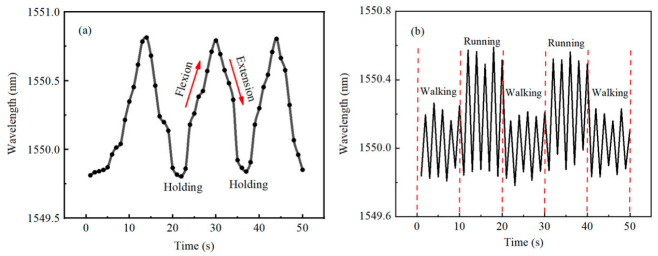
Knee joint motion detection. (**a**) The result of knee flexion and extension motion. (**b**) The result during walking and running processes.

Based on the above demodulation method, the detection results with bending and extension of the knee joint model are shown in Figure 9a. The figure illustrates the dynamic response of the FBG wavelength to periodic knee flexion and extension motion, as obtained through demodulation. The black line depicts the continuous variation in the FBG reflection wavelength within 50 s. A distinct and repeatable modulation is observed in the demodulated center wavelength of the FBG sensor, which is associated with the cyclical flexion and extension motion of the joint. The reflected wavelength of the FBG exhibits a significant and nearly linear increase (i.e., a redshift) when tensile strain is applied during knee flexion, and the wavelength increases from approximately 1549.81 nm to 1550.70 nm. Conversely, a marked decrease in wavelength occurs during the extension with the release of applied strain, returning the signal toward a stable initial baseline level. The reflection wavelength of the FBG shows a blueshift phenomenon because the extending process of the knee joint is equivalent to reducing the stretching strain of the FBG. During the experiment, the knee joint was repeatedly bent and extended. By tracking the complete change process of the FBG wavelength displacement, the movement state of the joint can be continuously and effectively monitored, especially during its cyclic transitions between bending, holding and extension. The smooth curves and stable baseline indicate an extremely low level of random noise in the measurement system. The sensor exhibits excellent repeatability, as evidenced by the two nearly identical peaks. The repeatability of the waveform shown in the figure further confirms the predominance of the effective signal. This provides a solid foundation for the accurate analysis of subsequent data and the credibility of the conclusion.

The FBG with PDMS was fixed on the knee joint of the volunteer, and the movement state of the joint was analyzed by monitoring the change in the reflection wavelength. A testing cycle of 10 s was set during which the volunteer performed walking or running. The FBG wavelength was demodulated every second, and the variation in the FBG reflection wavelength was analyzed accordingly during knee joint movement. By demodulating the FBG reflection wavelength corresponding to different optical power values, continuous tracking of joint movement was achieved over multiple testing cycles. The experimental results are shown in Figure 9b, which presents the demodulated FBG wavelength as a function of time over a 50 s interval, where the variation trend of the wavelength reflects the periodic motion of the knee joint. During walking, the central wavelength shifted by approximately 0.39 nm (from 1549.81 nm to 1550.20 nm), displaying regular low-amplitude oscillations. Conversely, running induced a larger shift of 0.79 nm (from 1549.81 nm to 1550.60 nm), characterized by high amplitude and frequency. This increase directly results from the greater strain in FBG experienced during joint motion. The feasibility of dynamic knee motion monitoring is evidenced by the clear differential response between the two locomotor states. Within 10 s for each state, approximately five complete oscillation peaks are observed, indicating an average joint cycle of 2 s. The waveforms remain in sync with the motion, further demonstrating the feasibility of dynamic monitoring. The results demonstrate effective knee joint motion detection and good repeatability of the sensor, thereby confirming the feasibility of the demodulation method. They indicate that the FBG can distinguish different levels of dynamic biomechanical loading based on wavelength shifts.

**Figure 10:**
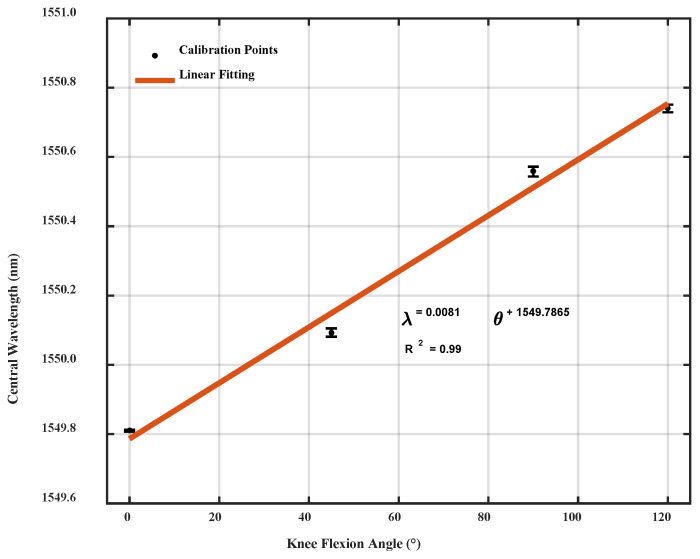
Central wavelength of the FBG versus knee flexion angle (0°, 45°, 90°, and 120°).

The relationship between the FBG central wavelength and knee flexion angle at 0°, 45°, 90°, and 120° is illustrated in Figure 10. This quantitative characterization elucidates the response of the FBG sensor to angular displacement. For each flexion angle, the mean wavelength and standard deviation were calculated from five repeated measurements, as represented by the data points and error bars, respectively. The solid line represents the linear fit to the data, yielding a coefficient of determination (R^2^ = 0.99), which indicates good linearity. The narrow distribution of error bars suggests good repeatability of the sensor. The calculated sensitivity is approximately 8.1 pm/°, confirming that the system can resolve small angular variations required for practical knee motion monitoring. The 0° angle corresponds to the initial state of the FBG and serves as the reference baseline. The result validates the dynamic trends observed in Figure 9, confirming the consistency between static calibration and dynamic measurement.

To clearly position the contribution of this work, the performance of the proposed method is quantitatively compared with existing representative demodulation techniques. While the OSA offers exceptional wavelength resolution and a wide dynamic range, its prohibitively high system cost and limited acquisition speed confine it to laboratory settings [29]. The tunable Fabry–Pérot filter achieves a good balance between resolution and range while offering a higher sampling rate, yet its system cost and complexity remain significant [33,39]. Although the matched FBG filter offers a lower-cost solution, its dynamic range is critically limited [34]. While the edge-filter method can operate at high speed and lower cost, it generally provides lower resolution and is susceptible to intensity noise [40]. In contrast, the method proposed in this work is essential for translating the spectral response of the sensors into quantifiable values of the underlying physical parameters. The method reduces the dependence on an optical spectrum analyzer and simplifies wavelength interrogation, while further integration is still required for portable applications. It offers a pragmatic alternative and facilitates the adoption of FBG technology for routine biomechanical monitoring.

In addition, although this work successfully demodulates a novel and low-cost method for joint motion detection, its performance is inherently constrained by fundamental noise, packaging reliability, and application scope. These limitations do not represent critical shortcomings but instead provide a clear roadmap for future research. Therefore, the robustness, intelligence, and practical utility of the system can be further enhanced by developing multiplexed demodulation algorithms, performing extensive clinical validation, and integrating more advanced signal processing and machine learning techniques. Future work will prioritize achieving synchronous multi-sensor demodulation in complex motion patterns and transitioning the technology into portable, low-power embedded health monitoring platforms.

## 4. Conclusions

In summary, a knee joint motion detection method based on the overlapping spectrum demodulation of an FBG and a tunable wavelength fiber laser is proposed. The FBG, encapsulated with PDMS, exhibits a good linear response with a sensitivity of 1.109 × 10^−3^ nm/με. This method converts complex spectral analysis into optical power measurements based on the overlapping spectrum demodulation of the FBG and fiber laser. The relationship between the optical power of the overlapping spectrum and the FBG wavelength shift induced by joint motion is theoretically derived and experimentally validated in the paper. The experimental results are fully consistent with the theoretical analysis, which further verify the feasibility of the proposed scheme and the excellent strain measurement performance of the system, including good linearity and good repeatability. The proposed joint motion detection setup has a simple structure and is suitable for practical applications, enabling convenient continuous monitoring and analysis of the knee joint movement. The quantitative comparison positions the performance of our system relative to prior art, while the discussion of its current constraints defines the scope for advancement. Together, they provide a solid foundation for assessing both the immediate contribution and the future potential of this technology. This study clarifies both the practical value and the future development path of the proposed technology, offering a comprehensive assessment for its translation toward biomedical applications. Future work will focus on extending this technology, with the aim of providing real-time clinical guidance for remote diagnosis, rehabilitation medicine, and sports health monitoring.

## Data Availability

Data will be made available on request.
